# Effect of plyometric versus complex training on core strength, lower limb, and upper limb power in male cricketers: a randomized controlled trial

**DOI:** 10.1186/s13102-023-00771-8

**Published:** 2023-11-27

**Authors:** Kamran Ali, Shubham Gupta, Mohammad E. Hussain, Msaad Alzhrani, Md. Dilshad Manzar, Masood Khan, Ahmad H. Alghadir

**Affiliations:** 1 Department of Physiotherapy, School of Medical and Allied Sciences, GD Goenka University, Sohna, Haryana India; 2 Sports Physiotherapy Division, Olympic Gold Quest, Delhi, India; 3 Allied Health Sciences and Physiotherapy, Shree Guru Gobind Singh Tricentenary University, Gurgaon, India; 4 Department of Physical Therapy and Health Rehabilitation, College of Applied Medical Sciences, Majmaah University, Majmaah, Saudi Arabia; 5 Department of Nursing, College of Applied Medical Sciences, Majmaah University, Majmaah, Saudi Arabia; 6 Department of Rehabilitation Sciences, College of Applied Medical Sciences, King Saud University, P.O. Box. 10219, Riyadh-11433, Saudi Arabia

**Keywords:** Muscle strength, Fitness, Athletes, Exercise, Power

## Abstract

**Background:**

Complex training is found effective in improving physical performance in various sports. There is a paucity of research evidence comparing the efficacy of complex vs. plyometric training in cricket players. The study aimed to compare the efficacy of complex and plyometric training on physical performance parameters in cricket players.

**Methods:**

Participants (*n* = 42 Male; age group = 18–26 years) were randomly allocated into three groups, complex training group (CTG) (*n* = 14; BMI = 20.51 ± 2.23), plyometric training group (PTG) (*n* = 14; BMI = 20.57 ± 2.82), and control group (CG) (*n* = 14; BMI = 20.51 ± 2.23). CTG and PTG received their respective training twice weekly, and CG received routine training for four weeks. Pre and post-intervention assessments of core muscle strength (CM), multistage fitness (MF), push-up (PU), lateral cone jump (LCJ), and stationary vertical jump (SVJ) were performed. This study has been registered in clinicaltrials.gov (ID: NCT05646914, on 05/12/2022).

**Results:**

A significant difference was observed between CTG vs. CG for CM (*p* ≤ 0.01), LCJ (*p* < 0.05), and SVJ (*p* ≤ 0.01), similarly in PTG vs. CG for CM (p-value), LCJ (*p* ≤ 0.05) and SVJ (*p* ≤ 0.01). However, No significant difference was found between PTG vs. CTG for any variables (*p* ≥ 0.05). Also, No significant difference in MF and PU was found between the groups (*p* ≥ 0.05).

**Conclusions:**

Complex training has been found to have effects similar to plyometric training alone. Therefore, either of the two strategies can be used to improve the performance of male cricket players.

## Introduction

Cricket is a team event that demands the players to assume different activities like throwing, bowling and batting, bowling, fielding, batting,.and wicket-keeping in the same game [1]. Due to its intermittent nature, this game imposes a considerable load on the physiological and neuromuscular systems [2]. It requires individuals to execute a range of movements with varying intensities, such as striding, sprinting, turning, and jumping. Muscle strength has been put forth as an important factor in enhancing the effectiveness of cricket-related activities, Bowlers benefit from upper body and leg strength to enhance their deliveries, batsmen rely on arm and core strength for powerful shots and balance, fielders utilize upper body strength for precise throws, and wicket-keepers depend on strong wrists and forearms for catching and stumping. Leg strength is crucial for running, striding, sprinting, making rapid turns, and jumping [2]. Improvements in strength gains have been observed following alterations in various dosage parameters of resistance training [3]. The frequency, duration, volume, follow-up period, order of exercises, and duration of rest have been regarded as the most important dosage variables used in resistance training [4].

Plyometric training has also gained popularity in recent years in enhancing athletes' strength and power output [5, 6]. This training comprises an eccentric muscle stretch followed by a concentric contraction and uses the stretch–shortening cycle [7]. This training stimulus has been found to produce significant increments in the vertical jump height and power output, even though it doesn’t require heavy external resistance [8].

Considering the isolated effectiveness of heavy and light resistance plyometric training for enhancing performance, a ‘complex training’ routine was devised by combining the aforementioned training forms [9]. Complex training starts with a high load of resistance training that is succeeded by plyometric training [10]. This combination aims at improving the efficacy of the plyometric training stimulus, thus increasing the neuromuscular response, explosive strength, and power [11, 12]. This response might further be improved by making appropriate variations in the dosage parameters of complex training. Previously available literature reports conflicting evidence, with some studies supporting the effectiveness of complex training for improving power output in sports [12–14], whereas others contradict this rationale [15].

Even though complex training effectively improves physical performance in various sports, there is a paucity of research evidence that directly compares the efficacy of plyometric and complex training for the outcomes of physical performance in cricket players. Thus, this study aimed to compare the effectiveness of complex and plyometric training for core muscle strength, power, and strength output in university-level male cricket players. It was hypothesized that complex training would significantly increase core muscle strength, power, and strength output compared to plyometric training and no treatment.

## Material and methods

### Study design, sample size calculation, clinical trial registration

**Fig. 1:**
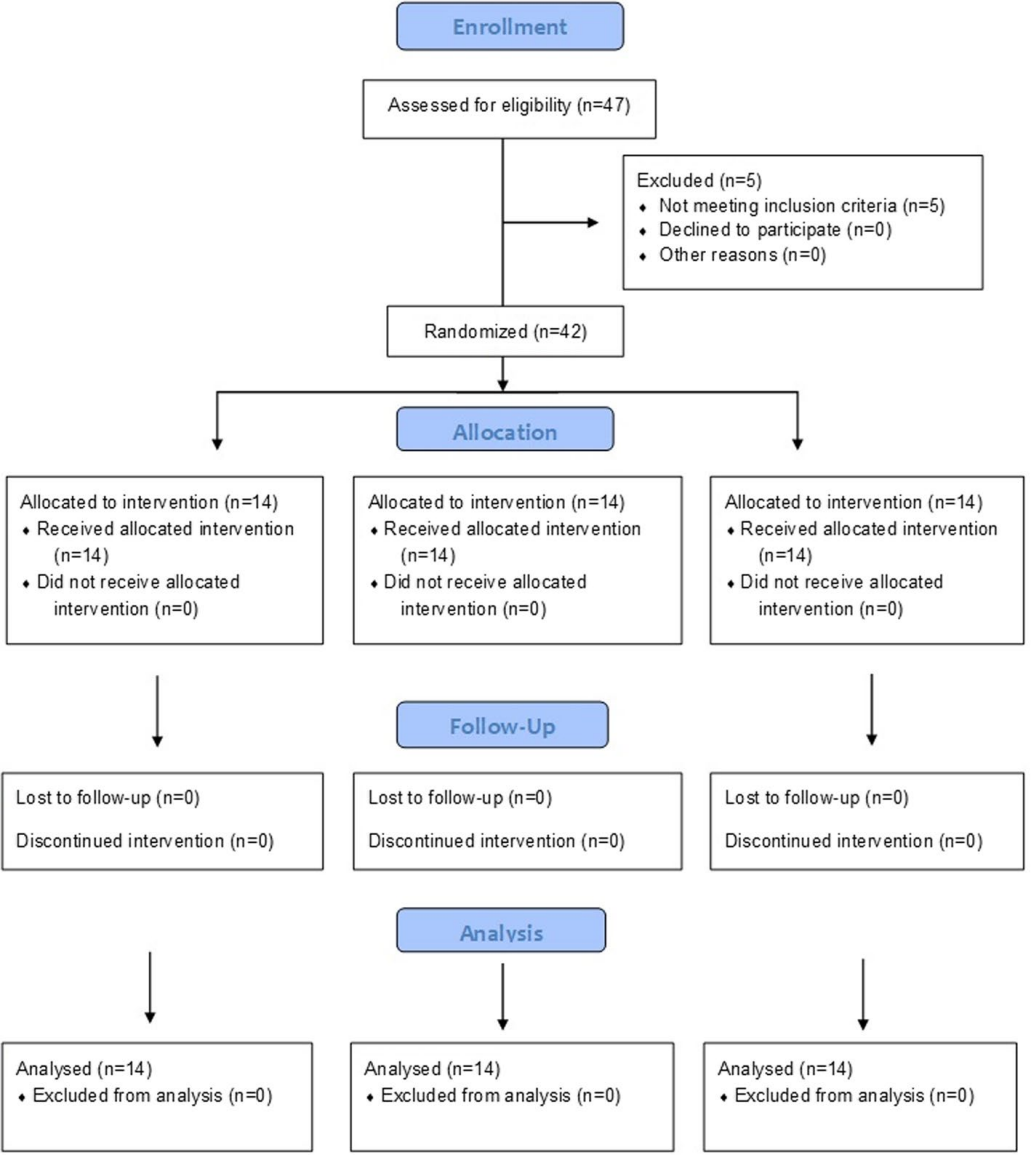
Consolidated Standards of Reporting Trials (CONSORT) flowchart of participants at each stage of the randomized trial

This was a randomized controlled trial with a pretest–posttest design. G Power v3.1.9.2 was used to calculate the sample size for this trial (*N* = 37) using effect size = 1.32; α = 0.05; power = 0.90 [16]. However, taking into account a dropout rate of 15%, we included 42 Male university-level cricket players, having at least 5–6 years of cricket playing experience, in this study in the age group of 18 to 26 years. Pre-intervention (or baseline) measures were taken after the familiarization period, and post-intervention readings were taken after four weeks of respective training [2, 17] (Fig. 1). All methods were performed in accordance with the relevant guidelines and regulations. The outcome assessor was kept blind to the allocation of participants. The participants were recruited from December 2015 to May 2016. The participants were recruited, and training was performed at the Centre for Physiotherapy and Rehabilitation Sciences, Jamia Millia Islamia, New Delhi, India. This study adhered to CONSORT guidelines for randomized controlled trials and had been registered on clinicaltrials.gov (ID: NCT05646914, on 05/12/2022).

Inclusion criteria for this study were as follows: Male subjects in the age group of 18 to 26 years, playing competitively at least once a month, involved in resistance training for at least six months, with working knowledge of the English language. Exclusion criteria were: a history of severe neurological deficit, injury or concussion in the past six months, operative treatment for lower or upper limb in the past six months, and current musculoskeletal pain (any level of chronicity). The demographic details of the participants in the three groups are summarized in Table 1.

**Table 1:** Comparison of Demographics and Outcome Measures at Baseline, *n* = 14 for each group

	CG (Mean ± SD)	CTG (Mean ± SD)	PTG (Mean ± SD)	F value	*p*-value
Age (yrs.)	19.7 ± 1.3	19 ± 1.4	19.5 ± 1.6	1.00	0.37
Height (cm)	176.7 ± 6.1	170.2 ± 7.3	174.9 ± 8.4	2.91	0.06
Weight (kg)	64.4 ± 10.2	55.8 ± 6.8	62.9 ± 11.0	3.18	0.05
BMI(kg/m^2^)	20.5 ± 2.2	19.1 ± 1.7	20.5 ± 2.8	1.70	0.19
CM Pre	124.2 ± 10.2	128.6 ± 8.7	124.2 ± 11.7	0.84	0.43
MF Pre	52.4 ± 9.4	59.4 ± 9.9	61.5 ± 15.7	2.20	0.12
PU Pre	33.6 ± 7.2	32.2 ± 10.9	32.6 ± 12.8	0.06	0.93
LCJ Pre	35.2 ± 5.3	36.5 ± 7.9	40.5 ± 8.9	1.84	0.17
SVJ Pre	16.2 ± 1.4	14.8 ± 1.8	16.1 ± 1.4	3.6	0.03*

*CG* Control group, *CTG* Complex training group, *PTG* Plyometric training group, *CM* Core muscle strength assessment, *MF* Multistage fitness assessment, *PU* Push-up assessment, *LCJ* Lateral cone jump assessment, *SVJ* Stationary vertical jump assessment, *SD* Standard deviation, *yrs* Year, *cm* Centimeter, *kg* Kilograms, *m* Meter

^*^Significant

A familiarization session was conducted to make the subjects aware of the testing and training procedures before the commencement of the study. A computer-generated random number table was generated, and subjects were randomly assigned into three groups, viz. control group (CG), complex training group (CTG), and plyometric training group (PTG). Randomization and allocation of participants were performed by an independent examiner not associated with the study. Each group comprised 14 participants. Independent assessments were carried out for the dependent variables at the baseline and following the completion of the training protocols. The outcome assessor was kept blind to the allocation of participants.

### The testing procedures for outcome measures

All subjects were instructed not to perform vigorous physical activity 24 h before the testing. We conducted three consecutive trials for each of the following performance tests and used the average of the measured variables for the final score:

1. **Core Muscle Strength Assessment** [18]: Athletes assumed a pike position with the elbows under the shoulders, the forearms shoulder-width apart on the floor, toes on the floor, buttocks in a neutral position, and the body in a straight line. The test was stopped if the hips were not in a straight line or any body part other than the forearm touched the ground. Measurement was done in seconds.
2. **Multistage Fitness Assessment** [19]: For this test, the subject had to run between two lines marked 66 feet apart (20 m). Pre-recorded beeps were played, and the subjects had to increase their speed with each beep, thereby determining their aerobic fitness based on the number of laps completed.
3. **Push-Up Assessment** [20]: The subjects performed the push-up with their hands and toes touching the floor. In the eccentric phase, the subjects moved down until their chest were at a 5 cm distance from the floor and back to its position till the elbow is fully extended. The total number of push-ups completed in proper form was counted. The test was stopped if the subjects could not maintain a neutral hip alignment or a complete range of motion.
4. **Lateral Cone Jump Assessment** [16]: Two cones were kept with a crossbar or tape fastened across them with the subject standing on one side. The subjects were made to jump to cross the bar and land on the **opposite** side. The time was started as soon as the subject’s foot left the ground, and the total number of lateral jumps performed in 30 s was counted. The test was stopped if the subject’s feet touched the tape barrier or knocked on the hurdle, and the total number of lateral jumps completed in 30 s was counted.
5. **Stationary Vertical Jump Assessment** [21]: A tape measure was attached to the wall, with the subject standing adjacent to it. They were supposed to touch the highest point on the wall with their inked middle fingertip. This point was taken as the standing height of the subject. Then, the subject performed a vertical jump with the assistance of both arms and legs to reach the maximum possible height and marked the wall with their inked middle fingertip. The difference between the two points was calculated as the final score.

### Training protocols

The control group (CG) performed routine training (not allowed to add new exercise), while the other two training groups (CTG and PTG) trained twice weekly for four weeks [2, 17]. Before initiating the training periods, the subjects in both groups were instructed about the proper execution of all exercises to be used during the training period for all training regimens. All of the training sessions were supervised. The weekly dosage and rest periods for complex training [9] and the plyometric training program are described in Table 2.

**Table 2:** Dosage characteristics of the plyometric and complex training group

	**Exercise**	**Week**	**Repetitions**	**Sets**	**Frequency**	**Intensity**	**Rest/Exercise**	**Rest/Set**
Plyometric training	Drop Jumps, Hops, Plyometric press-up, Box Jumps	I II III IV	10 10 8 8	2 2 3 3	2/wk	-	30 s/ 3 min	3 min
Complex training	Squats and drop jumps, Barbell step-ups and hops, Bench press and plyometric press-up, Barbell lunge and box jumps	I II III IV	8 8 6 6	2 2 3 3	2/wk	60% 70% 80% 90%	30 s/ 3 min	3 min

*wk* Weeks, *sec* Seconds

### Statistical evaluation

Statistical analysis was done using SPSS version 21.0 (SPSS Inc., Chicago, IL, USA). Data were assessed by a Shapiro–Wilk test for the normality of the distribution scores (*p* > 0.05). The demographic characteristics and the baseline criterion measures were compared among the three groups at the study entry by one-way analyses of variance (ANOVA). One-way ANCOVA was employed to determine the effect of different regimens on core muscle strength, multistage fitness, push-ups, lateral cone jump, and stationary vertical jump. The baseline readings were used as covariates, post-test assessments as dependent variables, and three exercise groups (CG, CTG, and PTG) as independent variables. As there was a significant difference in the baseline measure of stationary vertical jump among the three groups, therefore, the robustness of this outcome was further validated by performing One-way ANOVA using differences in stationary vertical jump (SVJ_pre_-SVJ_post_) as the dependent variable, and three exercise groups (CG, CTG, and PTG) as independent variable. This additional test reinforced the findings of One-way ANCOVA. A Bonferroni post hoc test was used to highlight the nature of any within and between-group differences. The significance level was set at *p* < 0.05.

## Results

All the participants (*n* = 42) were present for the post-intervention follow-up. All the descriptive baseline variables had non-significant differences among the three groups at baseline except stationary vertical jump height. A pre-test and post-test criterion measure conducted among the CG, CTG, and PTG showed significant improvements (time effect) in the core muscle strength, multi-stage fitness test, push-up, lateral cone jump, and stationary vertical jump measurements (*p* ≤ 0.001) (Ta). A significant difference was observed in the core muscle strength between the CTG vs. CG (*p* ≤ 0.01) and CG vs. PTG (*p* ≤ 0.05). A significant difference was observed in the lateral cone jump between the CG vs. PTG (*p* ≤ 0.05). Furthermore, a significant difference was also observed in the vertical jump height between the CG vs. CTG (*p* ≤ 0.01) and CG vs. PTG (*p* ≤ 0.01). No significant difference in multistage fitness and push-up assessment was found between the groups (*p* ≥ 0.05) (Table 3). A significant difference was also observed for lateral cone jump assessment but only between the control and complex training group. A significant time*group effect was demonstrated in the results for these participants (*p* ≤ 0.001). Pre-Post analysis of CG, CTG, and PTG for SVJ is shown in Table 3. Pre-Post analysis (mean ± SD) of Between-Group Comparison is shown in Table 3.

**Table 3:** Results (Mean ± SD) of Intervention Groups (plyometric and complex) and Control Group Before and After 4 Weeks

Variables	Group CG (Mean ± SD)	Group CTG (Mean ± SD)	Group PTG (Mean ± SD)	Time effect (*p*-value)	Time x Group effect (*p*-value)	Group effect (*p*-value)			
							**Pairwise comparison** **(** ***p*** **-value)**		
							**CG vs CTG**	**CTG vs PTG**	**PTG vs CG**
CM (Sec)									
Pretest	124.2 ± 10.2	128.6 ± 8.7	124.2 ± 11.7	< 0.001*	< 0.001*	0.005*	0.007*	1.00	0.030*
Post-test	123.2 ± 10.8	149.5 ± 12.8	148.5 ± 21.5						
MF (laps)									
Pretest	52.4 ± 9.4	59.4 ± 9.9	61.5 ± 15.7	< 0.001*	< 0.001*	0.070	0.224	1.00	0.092
Post-test	53.2 ± 9.1	62.8 ± 10.2	64.4 ± 15.6						
PU (reps)									
Pretest	33.6 ± 7.2	32.2 ± 10.9	32.6 ± 12.8	< 0.001*	< 0.001*	0.915	1.00	1.00	1.00
Post-test	34.2 ± 6.9	37.6 ± 11.7	38.7 ± 14.8						
L C J (reps)									
Pretest	35.2 ± 5.3	36.5 ± 7.9	40.5 ± 8.9	< 0.001*	< 0.001*	0.040*	1.00	0.318	0.038*
Post-test	36.2 ± 6.3	40.4 ± 8.4	46 ± 8.2						
S V J (cm)									
Pretest	16.2 ± 1.4	14.8 ± 1.8	16.1 ± 1.4	< 0.001*	< 0.001*	< 0.001*	< 0.001*	0.184	< 0.001*
Post-test	16.1 ± 1.0	16.5 ± 1.7	18 ± 1.2						

*CG* Control group, *CTG* Complex training group, *PTG* Plyometric training group, *CM* Core muscle strength assessment, *MF* Multistage fitness assessment, *PU* Push-up assessment, *LCJ* Lateral cone jump assessment, *SVJ* Stationary vertical jump assessment, *SD* Standard deviation, *sec* Seconds, *reps* Repetition

^*^Significant

## Discussion

To the best of our knowledge, it was the first study that assessed the comparative effectiveness of plyometric and complex training procedures in core strength, lateral jump, and vertical jump performance in male cricket players. This study demonstrated an increase in core muscle strength and vertical jump height following complex training. Similarly, plyometric training also showed significant improvements in core strength, lateral cone jump, and stationary vertical jump compared to the control group. Furthermore, group comparison analysis indicated more improvement in the complex training group's vertical jump than in the plyometric training group. However, Complex training produced almost similar improvements compared to plyometric training in core strength, multistage fitness, push-up, and lateral cone jump tests. This signifies that complex training is equally beneficial for upper limb performance and produces similar improvements in aerobic fitness when compared with plyometric training alone.

Previous studies supported the use of alternating high and low-load exercises [22]. The improvements observed in vertical jump performance following the complex training might be attributed to the improvements in neural adaptations such as enhanced higher motor center activity, myoelectrical potentiation, synchronization of motor unit firing rate, reduction of inhibition from Golgi Tendon Organ and Renshaw cells, and reciprocal inhibition of antagonist muscle groups [23, 24].

In coherence with our study, previous researchers [25] reported that heavy dynamic resistance exercise led to non-significant improvements in maximal strength and power output during power push-ups. Baker et al. showed that a 65% load effectively produced increments in upper limb power output [11]. The use of a higher load might be a reason for the non-significant improvements observed in our study. This suggests that a higher load of 80–85% might not be required for the smaller upper extremity muscles, and an optimal load should be used for training these muscles. The smaller muscle mass of the upper extremity might be overloaded as a higher load is placed on the muscle–tendon unit, thereby precluding significant improvements in these muscle groups. However, the 85–90% load was found to be effective in improving the lower limb power output supporting the rationale of a higher resistance load for the larger lower leg muscles. The improvements observed in lower extremity muscles align with previous literature that suggests a high load in resistance training is better than a low load at improving muscle size and strength in sedentary men [26]. Another factor for a non-significant improvement in the upper extremity might be attributed to shorter rest duration and limited number of exercises for upper extrimety in this study compared to previous literature. Evans et al. [27] found a statistically significant effect of a bench press complex training on medicine ball throw with a four minutes rest period compared to three minutes in this study. In contrary, Ritchie D et al. [28] found Utilizing 1-min recovery intervals could be an efficient way to prescribe strength-power exercises compared to different loading schemes, Extended recovery periods do not seem to enhance immediate subsequent performance. A shorter rest duration might not allow the smaller upper extremity muscles to recover from loads of the previous exercises, leading to higher fatigue and reduced improvement [27]. This decrease might be attributed to a reduction in muscle’s anabolic responses owing to a decrement in muscle protein synthesis because of shorter rest intervals [29]. However, the substrate recovery is higher in the case of larger leg muscles. 70% of the substrates are recovered within 30 s, with complete recovery in three to five minutes. This heightened recovery of substrates in the larger muscle groups might improve lower leg muscles in the complex and plyometric training groups [30].

So, there is mixed evidence of the effectiveness of plyometric and complex training. Some studies support the positive effects of plyometric training, whereas some showed equal effectiveness of the two types of training [13, 30]. One of the studies suggested an improvement in muscle strength following a combined intervention, including weight and plyometric training [13]. Previous literature also suggests using a combination of both weight training and plyometric training rather than using either of the two strategies [31]. On the contrary, Herrero et al. showed that a plyometric protocol could not significantly improve vertical jump height [32].

The non-significant improvements observed in the upper limb performance variable indicate that complex training is not superior to plyometric training alone for improving upper limb performance in male cricket players. But complex training can be an ideal strategy for improving lower limb strength and power. However, certain limitations restrict the broad applicability of the study's findings. The study predominantly focuses on lower limb exercises, with fewer upper limb exercises included in the protocol. Moreover, the study's emphasis on male participants aged 18–26 limits the ability to generalize the results to other populations. The relatively small sample size (42 participants, with 14 in each group) raises concerns about statistical power and the potential for biases. The short intervention duration may not capture long-term benefits, and uncontrolled factors such as individual fitness levels and prior training experiences could confound the results. Additionally, overlooking variables related to nutrition, diet, and perceived exertion may influence participants' responses. Future research should encompass a more diverse and extensive participant pool, longer intervention periods, the control of individual factors, and consideration of dietary and perceptual aspects. Addressing these issues will yield a more comprehensive understanding of training strategies for cricket players and their practical implications for sports professionals.

### Practical recommendations

Incorporating plyometric and complex training within the sports player's program is an effective means to optimize their strength, power, and overall performance. These methods significantly contribute to enhancing athletic capabilities, offering important insights for professionals like athletic trainers, strength and conditioning experts, and physical therapists tasked with designing tailored training regimens to unlock the full potential of cricket players.

## Conclusion

Study revealed significant improvements in core muscle strength and jump-related measurements (lateral cone jump and vertical jump) following complex and plyometric training. However, there were no significant differences in multi-stage fitness and push-up assessments between the groups. These findings offer valuable insights for enhancing athlete performance and training strategies.

## Data Availability

The data associated with the paper are not publicly available but are available from the corresponding author on reasonable request.
